# Early outcomes after focal high-dose-rate brachytherapy in low- and favorable intermediate-risk prostate cancer: promising early results from a prospective randomized trial

**DOI:** 10.1515/med-2026-1450

**Published:** 2026-07-20

**Authors:** Justinas Jonusas, Ausvydas Patasius, Mantas Trakymas, Kestutis Akelaitis, Mindaugas Dziugelis, Marius Burkanas, Jonas Venius, Giedre Smailyte, Marius Kincius

**Affiliations:** Clinic of Hematology and Oncology, Institute of Clinical Medicine, Faculty of Medicine, Vilnius University, Vilnius, Lithuania; Laboratory of Cancer Epidemiology, National Cancer Institute, Vilnius, Lithuania; Department of Public Health, Institute of Health Sciences, Faculty of Medicine, Vilnius University, Vilnius, Lithuania; Center of Diagnostic Oncology, National Cancer Center, Vilnius, Lithuania; Center of Radiation Oncology, National Cancer Center, Vilnius, Lithuania; Department of Oncourology, National Cancer Center, Vilnius, Lithuania

**Keywords:** prostate cancer, focal therapy, high-dose-rate brachytherapy, active surveillance, randomised control trial

## Abstract

**Objectives:**

To compare the QoL of patients diagnosed with low- or favorable-intermediate-risk PCa after focal HDR brachytherapy and active surveillance. Our secondary objectives were to compare progression-free survival (PFS) between patients who underwent focal treatment and those who were actively surveilled and to assess early and late GU and GI adverse effects after the focal HDR brachytherapy.

**Methods:**

This prospective randomized controlled trial was conducted at the National Cancer Center located in Vilnius, Lithuania. The study was initiated after approval from the Vilnius Regional Biomedical Research Ethics Committee was obtained (Approval ID 2022/6-1438-911). Twenty-seven patients were assigned to the AS group and 28 to the focal HDR group.

**Results:**

During the median follow-up time of 14 and 17 months for AS and HDR groups, respectively, the QoL, evaluated by the EORTC QLQ-30 and PR25 instruments, showed no statistically significant differences in functional scores between the groups after baseline adjustment. Additionally, patients in the HDR group reported better urinary outcomes as measured by EORTC PR25 and IPSS scores at 6, 12, and 18 months. There were no statistically significant differences in erectile function between the groups as measured by the IIEF. Early toxicity was low (no Grade ≥3 events), and only two cases (7.1 %) of Grade 2 GU toxicity were reported in the HDR group within the first 3 months, with one case persisting for 6 months. Late toxicity was not assessed during this interim follow-up. The HDR group demonstrated a non-significant trend toward better oncological outcomes, with fewer PSA-based recurrences and a higher mean PFS than the AS. At the time of last follow-up, 49/55 (89 %) patients were censored (22/27 in the AS and 27/28 in the HDR group).

**Conclusions:**

A current study shows comparable QoL and early toxicity results between AS and HDR groups. Any domain-level signals were below MCID and did not persist after baseline adjustment. Results of PFS are underpowered at this interim analysis due to short follow-up and high censoring, and should be considered hypothesis-generating only. However, as current results are only exploratory and insufficient to assess durable disease control or late toxicity, a longer follow-up, larger cohorts, and a multicenter approach are needed to provide definitive outcomes to demonstrate the benefit of focal brachytherapy.

## Introduction

Prostate cancer (PCa) retains its position as one of the most prevalent malignancies among men worldwide [1]. The widespread adoption of prostate-specific antigen (PSA) testing allowed the detection of PCa at early stages, permitting the timely use of radical treatment [2]. However, concerns were raised regarding the overtreatment and the decrease in quality of life (QoL) associated with the significant morbidity that comes after the radical treatment in the form of erectile dysfunction, urinary incontinence, and bowel symptoms [3], 4].

In order to reduce the overtreatment and preserve the QoL among men diagnosed with low- and selected cases of intermediate-risk PCa, active surveillance (AS) has been introduced as a disease management option. The long-term results, with follow-up data of up to 15 years from the PIVOT, SPCG4, and ProtecT trials, demonstrated that there are no statistically significant differences between the AS and radical treatments for these patients [5], 6]. However, some of the patients under the AS eventually have to be treated either because of the disease progression or the mental distress that patients are experiencing while living with the untreated PCa. Thus, focal therapy has emerged as a middle ground combining the treatment of the dominant lesion and the surveillance of the untreated prostate while preserving its functions and reducing the prevalence of complications associated with the radical whole gland treatment [7], [8], [9].

Among all focal treatment modalities available for localized PCa, focal brachytherapy is of particular interest due to its ability to deliver highly conformal radiation doses to the lesion. Early feasibility studies, including high- (HDR) and low-dose-rate (LDR) brachytherapy, showed promising results with biochemical relapse-free survival ranging from 70 to 100 % during 20–72 month follow-up periods [7], [10], [11], [12], [13], [14], [15], [16]. Moreover, findings demonstrate that focal brachytherapy, using both HDR and LDR techniques, is associated with relatively low genitourethral (GU) and gastrointestinal (GI) toxicities. Usually, only grade 1 GI toxicities are observed, and no significant long-term side effects have been reported. Temporary urinary symptoms, such as frequency and retention, peak at 2 months, returning to baseline by 6 months. Moderate worsening of erectile function post-treatment is observed, with partial recovery by 6–12 months in the majority of studies. These results are well summarised in recent systematic reviews. In a meta-analysis by Gutierrez-Valencia et al., which included 1,492 patients, 14 studies reported on focal brachytherapy as monotherapy, with biochemical control at 24 months reaching 97 % (95 % CI [86–99 %]) and 82 % (95 % CI [65–92 %]) at 60 months [9]. Similar results were reported in other meta-analysis by Mohamad et al. [8]. Pooling 10 studies with 315 patients included, a definitive focal BT demonstrated a 91 % (95 % CI [82–95 %]) biochemical recurrence-free survival at 4 years. The GU and GI events were predominantly low-grade (acute G2 or less toxicities: GU 7 %, GI 3.5 %. Late G2 or less toxicities: GU 2 %, GI 4.4 %). No grade 3 or higher toxicities were reported. This is particularly significant as it indicates a reduced risk of side effects for patients undergoing focal brachytherapy as a treatment option for low- to intermediate-risk localized prostate cancer.

However, there is a lack of evidence about the change in the quality of life (QoL) after focal treatment and its comparison with AS from randomized controlled trials (RCT), as both disease control options are available to patients diagnosed with low- and favorable-intermediate-risk PCa. To address this, we initiated a prospective RCT comparing QoL in the cohort of patients diagnosed with low- or favorable-intermediate-risk PCa who underwent focal HDR brachytherapy (BT) or active surveillance. Our secondary objectives were to compare progression-free survival (PFS) between patients who underwent focal treatment and those who were actively surveilled and to assess early and late GU and GI adverse effects after focal HDR brachytherapy. We hypothesize that focal HDR BT would preserve QoL compared to AS while maintaining a low toxicity profile.

## Materials and methods

This prospective randomized controlled trial was conducted at the National Cancer Center (NCC) located in Vilnius, Lithuania. NCC is a high-volume academic center with an established focal brachytherapy program. Treatment and surveillance followed standard operating procedures described in the quality assurance documents of NCC with multidisciplinary review. The original protocol of the RCT was published elsewhere [17].

Males 40–75 years old and diagnosed with low or favorable intermediate-risk PCa (PSA≤10 ng/mL, ISUP≤2, cT1 – T2b, Index lesion ≥0.5 cm^3^, IPSS≤18) in our institution were invited to participate in the ongoing RCT. Exclusion criteria were prior definitive treatment of prostate, T3 disease with extracapsular extension, and failure to sign an informed consent form.

According to the protocol, patients are randomized into one of three groups (HDR BT, LDR BT, and AS) with a ratio of 1:1:1. Due to poor accrual of patients in the focal LDR brachytherapy arm, results from this group are not presented in the current analysis. However, as RCT continues to accrue patients, the randomisation scheme has not been adjusted, and long-term results are expected to represent a complete dataset as expected during RCT design.

– Focal HDR BT group:

For patients randomized into this group, a fraction of 19 Gy was delivered using an iridium-192 source under general anesthesia. Diagnostic 1.5 T multi-parametric magnetic resonance images (mpMRI) were fused with real-time ultrasound (US) images to delineate the focal clinical tumor volume (fCTV), which was expanded by a 5 mm isotropic margin to form a focal planning target volume (fPTV).

– Active surveillance group:

Patients randomized to this control group received no treatment and were surveilled by the active surveillance algorithm approved by our center.

The primary endpoint of the ongoing RCT is the QoL of patients, which is assessed using the European Organisation for Research and Treatment of Cancer (EORTC QLQ-C30) and prostate cancer-specific (PR25) questionnaires. The urinary function was evaluated using the International Prostate Symptom Score (IPSS), while erectile function was evaluated using the International Index of Erectile Function score (IIEF). All questionnaires were filled at the baseline and during the follow-up visits (every 6 months). Additionally, the QoL instruments were administered in the local language, and the validated translations were used.

The secondary endpoint was PFS, which was defined as the time from randomization to clinical (negative changes in the control mpMRI or biopsy (targeted MRI/US fusion)) or biochemical disease progression (two observed PSA increases in a row). Clinical assessments included the measurement of PSA levels and collecting urine samples at baseline and every 6 months, performing a control mpMRI 9–12 months after randomization, and performing targeted MRI/US fusion biopsy afterward. Additionally, early and late GI and GU toxicities were recorded using RTOG criteria at 1, 3, and 6 months and then every 6 months thereafter.

All statistical calculations were performed using the IBM SPSS Statistics package (Armonk, NY: IBM Corp). For a continuous variable, we calculate the mean (standard deviation (SD)) or otherwise the median (Q1, Q3). For discrete variables, we present the median with minimum and maximum values. The normality assumption was checked using the Shapiro-Wilk test. For normally distributed variables, we use a *t*-test to compare means and the Mann-Whitney *U* test for median comparison when the distribution is non-normal. For normally distributed variables, we use a one-way ANOVA to test for differences in means when comparing more than two independent groups. The Turkey criterion was used for pairwise comparisons. For non-normally distributed variables, we used the Kruskal-Wallis test to compare medians between three or more independent groups. The QLQ30 and PR25 domains were analysed using linear mixed-effects models, and no imputation was performed. The baseline scores and T-stage were used as covariates for baseline adjustment and for a sensitivity analysis accordingly. Additionally, as the trial is still actively enrolling, later follow-up time points include administrative missingness rather than withdrawal for participants who have not yet reached the planned visits. Finally, no formal adjustment for multiple comparisons was performed across QoL domains and time-points. Thus, the significance of isolated differences should be regarded as exploratory and interpreted with caution.

The frequency distributions between groups are compared with the Chi-square or Fisher’s exact test based on the expected frequencies. The patients were censored at the last follow-up date if there was no PFS. Survival trends and median (95 % confidence interval [CI]) survival were analyzed using the Kaplan-Meier method. Differences between survival curves were evaluated using the Log-rank test. Additionally, TNM-adjusted sensitivity analysis for PFS was performed. The Cox model stratified by baseline TNM stage was used because of the small number of events, with treatment group entered as the sole covariate.

The study was approved by the Vilnius Regional Biomedical Research Ethics Committee (Approval ID 2022/6-1438-911). The study was conducted in accordance with the Declaration of Helsinki (as revised in 2013). Informed consent was obtained from all individuals included in this study, or their legal guardians or wards.

## Results

A total of 55 patients were enrolled in the study over the two-year period from the initiation of the study in 2023. Twenty-seven patients were assigned to the AS group and 28 to the focal HDR group. Baseline characteristics between the groups were comparable (Table 1). The mean age at diagnosis was 66.2±7.0 and 63.8±7.7 years in the AS and HDR groups, respectively (p=0.232). Median follow-up duration was also similar: 14 [6–36] months for AS and 17 [6–33] months for HDR (p=0.337). There was no significant difference in initial PSA levels between the two groups (5.23±2.33 ng/mL vs. 5.74±1.98 ng/mL, p=0.143). A higher proportion of patients in the HDR group were diagnosed with ISUP Grade Group 2 (35.7 %) PCa compared to 11.1 % in the AS group. However, the difference was not statistically significant (p=0.055). 92.9 % of HDR patients had T1 disease compared to 70.4 % in the AS group (p=0.040). PIRADS distribution and mean prostate volume did not differ significantly between groups (p=0.240 and p=0.470, respectively).

**Table 1:** General data of patients involved in the trial.

Number of patients	55		
AS	27		
HDR	28		

	**AS**	**HDR**	**Significance**

Mean age at diagnosis, years	66.19±7.02	63.79±7.66	0.232
Median follow-up time, months	14 [6–36]	17 [6–33]	0.337
Mean starting PSA, ng/mL	5.23±2.33	5.74±1.98	0.143
Grade group			
1	24 (88.9 %)	18 (64.3 %)	
2	3 (11.1 %)	10 (35.7 %)	0.055
TNM stage			
T1	19 (70.4 %)	26 (92.9 %)	
T2	8 (29.6 %)	2 (7.1 %)	**0.040**
PIRADS score			
2	0 (0.0 %)	1 (3.6 %)	
3	2 (11.1 %)	0 (0.0 %)	
4	18 (66.7 %)	21 (75.0 %)	
5	6 (22.2 %)	6 (21.4 %)	0.240
Mean prostate volume, mL	52.82±19.95	48.98±18.81	0.470

The bolded value (0.040) represents a statistically significant difference, as p<0.05.

**Figure 1:**
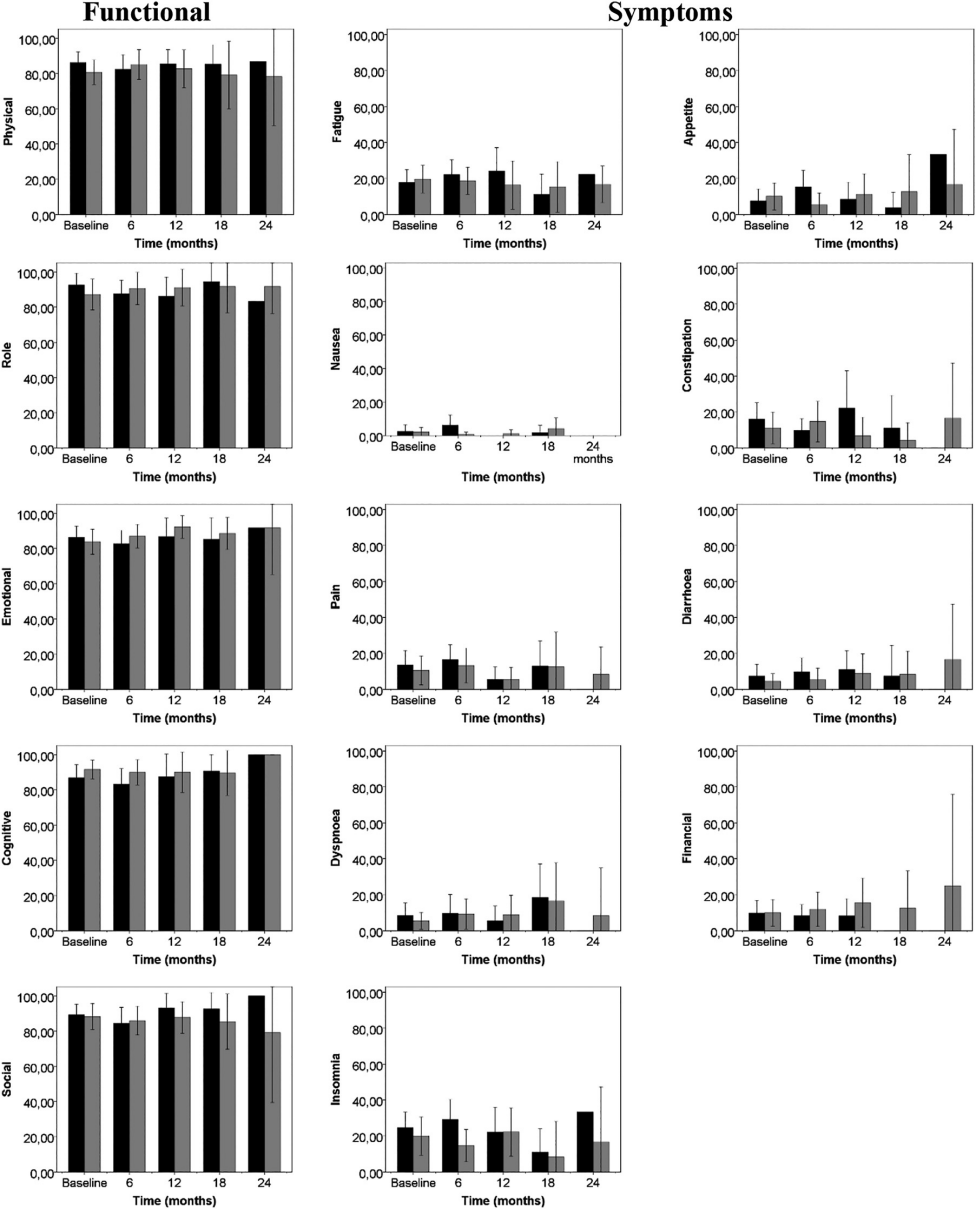
EORTC QLQ30 questionnaire results during the follow-up time. Results are presented as means with 95 % confidence intervals. Grey and black bars represent the HDR and AS groups, respectively.

First, the quality of life was evaluated between the groups by analyzing the results of the EORTC QLQ30 questionnaire (Figure 1). Firstly, in unadjusted models, nausea, insomnia, and appetite domains showed a non-significant trend at the 6-month time point (6.17±14.54 vs. 0.67±3.34, p=0.043, 15.21±21.88 vs. 5.33±15.76, p=0.069, and 29.08±26.49 vs. 14.62±21.61, p=0.064). However, in baseline-adjusted mixed models, no statistically significant differences were observed across QLQ-C30 domains, and those trends were not sustained (p>0.05). It is worth mentioning that questionnaire completion decreased at later visits primarily due to staggered entry. The per-time-point questionnaire completion rates are shown in Table 2.

**Table 2:** Completion rates by arm and follow-up time are presented, with the number of participants who reached each time point at the data cut-off is shown.

Group	Baseline	6 months	12 months	18 months	24 months
**AS**	27	27	12	9	3
**HDR**	28	28	15	8	4

**Figure 2:**
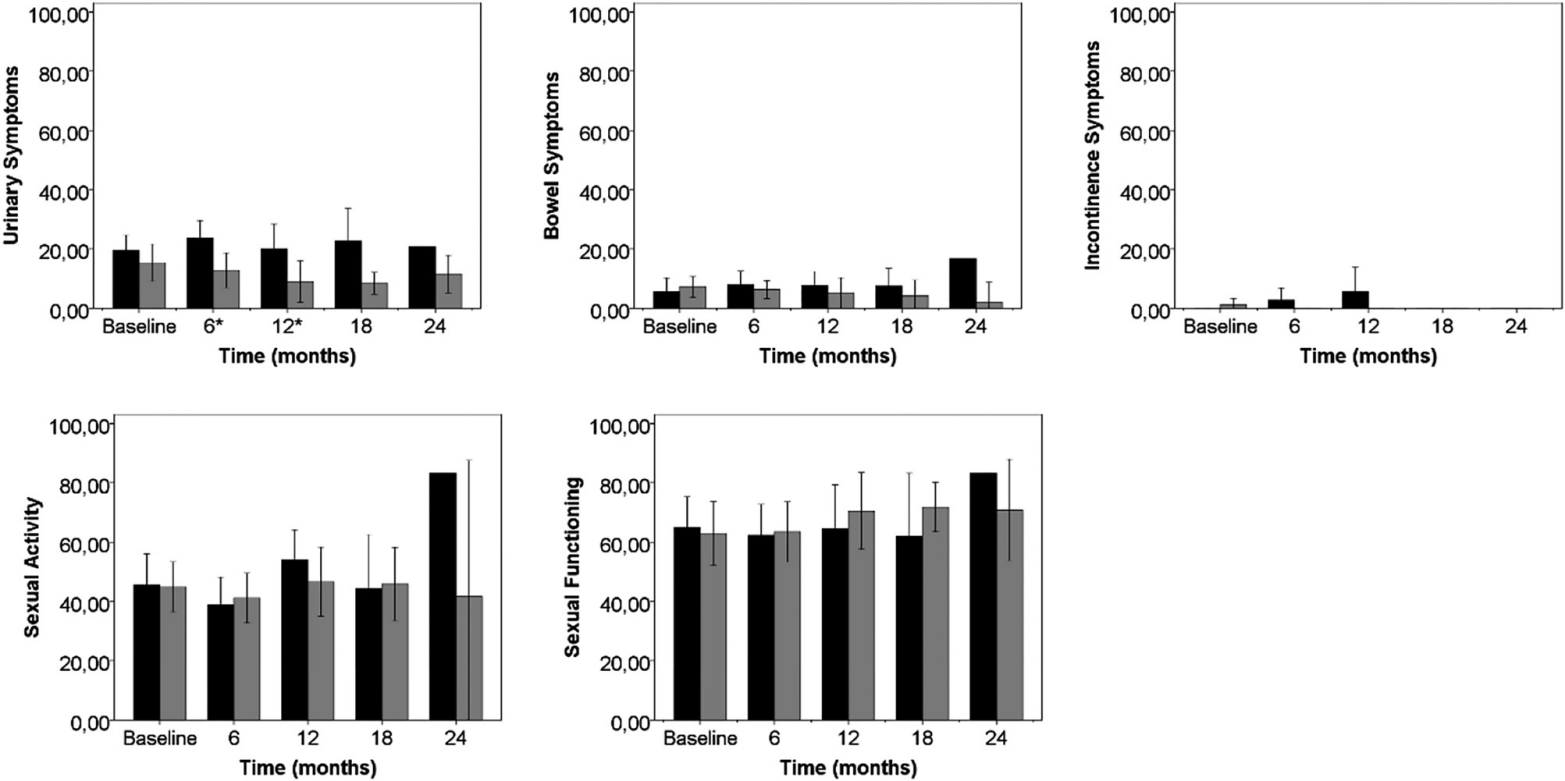
EORTC PR25 questionnaire results during the follow-up time. Results are presented as means with 95 % confidence intervals. Grey and black bars represent the HDR and AS groups, respectively. *Statistically significant (p<0.05) difference between the AS and HDR groups at a given time. However, p-values are not adjusted for multiple comparisons across domains or time points, thus the results are only exploratory.

There was no statistically significant difference between the AS and HDR groups when the EORTC PR25 questionnaires were analyzed, except for urinary symptoms (Figure 2). Interestingly, patients randomized to the AS group experienced higher urinary symptoms at 6 and 12 months during follow-up than those treated (23.64±14.04 vs. 12.71±14.28, p=0.005 at 6 months and 20.01±13.17 vs. 8.88±12.59, p=0.008 at 12 months).

**Figure 3:**
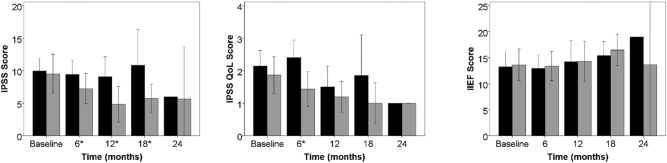
IPSS and IIEF questionnaire results during the follow-up time. Results are presented as means with 95 % confidence intervals. Grey and black bars represent the HDR and AS groups, respectively. *Statistically significant (p<0.05) difference between the AS and HDR groups at a given time.

Similarly, there was a statistically significant difference between groups in IPSS scores at 6, 12 and 18 months, with the AS group having higher scores (9.43±4.92 vs. 7.24±5.65, p=0.048 at 6 months, 9.08±4.83 vs. 4.87±4.93, p=0.012 at 12 months and 10.86±5.96 vs. 5.75±6.50, p=0.049 at 18 months), although no statistically significant difference was observed at baseline (Figure 3). Additionally, patients in the AS group demonstrated lower IPSS QoL at a 6-month time point (2.41±1.22 vs. 1.44 vs. 1.29). There was no difference between the groups in erectile function, as assessed by the IIEF scale.

As a secondary outcome, early and late GU and GI reactions were evaluated using RTOG guidelines (Table 3). Early GU reactions were scarce and limited to grade 2 (2 patients (7.1 %) at 1 month had moderate dysuria and increased frequency. For one patient (3.6 %), reactions remained for 6 months). Early GI reactions were rare, with only one patient (3.6 %) experiencing mild rectal discomfort for 3 months after the treatment. There were no late grade 2 GU or GI reactions, and only one patient experienced a mild decrease in the urinary stream at 6 months and later.

**Table 3:** Number (percent) and grade of GU and GI reactions reported during the follow-up period.

Grade	1 month		3 months		6 months		12 months		18 months		24 months	
	GU	GI	GU	GI	GU	GI	GU	GI	GU	GI	GU	GI
1	4 (14.3)	1 (3.6)	4 (14.3)	1 (3.6)	2 (7.1)	0 (0)	1 (3.6)	0 (0)	1 (3.6)	0 (0)	0 (0)	0 (0)
2	2 (7.1)	0 (0)	2 (7.1)	0 (0)	1 (3.6)	0 (0)	0 (0)	0 (0)	0 (0)	0 (0)	0 (0)	0 (0)
Total^a^	28		28		28		15		8		4	

^a^Shows a total number of subjects who reached a specific follow-up time point.

**Figure 4:**
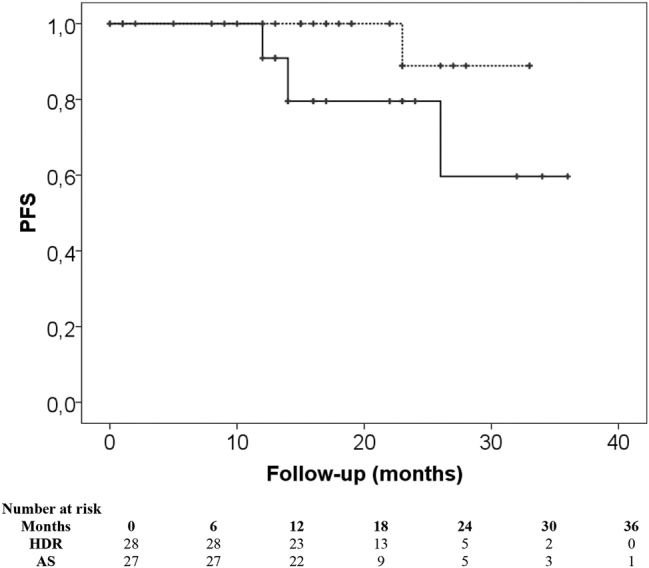
Kaplan–Meier function on PFS. The dotted line represents the HDR group, while the solid line represents the AS group. Median follow-up time was 14 [6–36] and 17 [6–33] months for AS and HDR groups, respectively. Given the high censoring rate, later time-point survival estimates are unstable and should be interpreted cautiously.

Finally, PFS was analyzed using the Kaplan-Meier method (Figure 4). There were five (18.5 %) progressions in the AS group and only one (3.6 %) in the HDR group. The remaining subjects were censored at the last follow-up. Patients randomized to the AS group had a mean PFS of 29.33 months (95 % CI [24.34–34.32]), while the mean PFS in the HDR group was 31.89 months (95 % CI [29.83–33.94]). The survival distributions for the AS and HDR groups showed a non-significant trend with the potential for HDR to have longer PFS (χ2 (2) = 3.361, p=0.067). The hazard of progression was consistent in direction, but also statistically insignificant, with an HR of 0.18 (95 % CI [0.02–1.62]) in the HDR group in a TNM-stratified Cox model. However, given the limited number of events, the interim data cut, and the high censoring rate, these PFS comparisons are exploratory and should be interpreted with caution, as later time-point survival estimates may be unstable.

## Discussion

The main findings of our ongoing RCT, comparing focal HDR BT with AS in patients with low- and intermediate-risk PCa, were that QoL, evaluated by the EORTC QLQ-30 and PR25 instruments, showed no statistically significant differences in functional scores between the groups during the follow-up time. However, patients in the HDR group reported better urinary outcomes as measured by EORTC PR25 and IPSS scores at 6, 12, and 18 months. There were no statistically significant differences in erectile function between the groups as measured by the IIEF. Moreover, toxicity profiles were favorable, with early GU and GI toxicities being rare. There were no Grade 3 or higher events observed, and only two cases of Grade 2 GU toxicity were reported in the HDR group within the first 3 months, with one case persisting for 6 months. No significant late toxicities were documented. Finally, in terms of PFS, the HDR group demonstrated a non-significant trend toward better outcomes, with fewer PSA-based recurrences and a higher mean PFS compared to the AS group. However, the PFS results are exploratory and should be interpreted with caution.

Only one study has used the EORTC-QLQ30 and PR25 questionnaires to evaluate QoL after focal BT. In a study by Maenhout et al., there was no statistically significant decrease in EORTC-QLQ30 and PR25 scores after the focal HDR BT during the follow-up period [18]. However, the authors observed a clinically relevant, although statistically nonsignificant, decrease in fatigue, cognitive, and general health scores in the QLQ30, as well as a decrease in sexual activity, as indicated by the PR25 questionnaire, at later time points (a decrease of more than 10 points from baseline). Our results are comparable, showing a stable QoL during the follow-up period in the HDR group (Figure 1). Interestingly, patients randomized to the AS group experienced statistically significantly higher urinary symptoms than those in the HDR group at 6 and 12 months. This could be explained by the psychological or symptomatic burden associated with active surveillance [19], 20]. On the other hand, these results should be interpreted carefully. Although several QoL domains showed a non-significant trends, the absolute differences were small and below the commonly cited minimal clinically important difference. This indicates a limited clinical relevance. Additionally, given the short follow-up time, we treat these findings as exploratory, and we will re-evaluate these results with the longer follow-up and the full three-arm dataset. Moreover, interpretation of outcomes should consider the baseline T-stage imbalance, with more T1 disease in the HDR group. While baseline-adjusted urinary analyses yielded similar findings, the possibility of residual confounding remains.

Interestingly, patients treated with focal HDR brachytherapy showed statistically significantly better urinary function compared to those in the AS group at 6, 12, and 18 months, when it was evaluated using the IPSS score (Figure 3). However, when the IPPS score was compared within groups, no statistically significant difference was observed. Our results are in agreement with those published in other focal brachytherapy papers. Graff et al., Hass et al., and Prada et al. reported no statistically significant change in IPSS score after the focal BT procedure [11], 12], 16]. Meanwhile, Peters et al., Ta et al., and Langley et al. showed a temporary increase in IPSS score after focal treatment, which returned to the baseline level shortly during follow-up [10], 15], 21]. Our findings, showing that patients treated with focal HDR had a lesser urinary burden than their counterparts in the AS group, may be explained by the idea that subclinical obstructive symptoms associated with the index lesion were relieved after targeted irradiation. Additionally, as benign prostate hyperplasia (BPH) was not recorded during the investigation, obstructive symptoms related to it proceeded unmodified in the AS group. Meanwhile, alpha-blockers that were prescribed to the HDR group after the procedure may transiently benefit this group, resulting in better outcomes. In contrast, erectile function remained comparable between the AS and HDR groups during follow-up in our trial, as measured using the IIEF questionnaire. These results are similar to those reported in previous papers, where no changes in IIEF scores were observed, or only mild declines were observed after the focal BT [11], 12], 15], 21].

A published paper by Prada et al. showed no chronic urinary toxicity and only a mild decrease in IPSS from 8.2 at baseline to 6.7 at 24 months, which was not statistically significant during the follow-up time [11]. As for rectal toxicity, no early or late adverse events were reported. Comparable results were published by Peters et al., where the authors showed no late GU or GI toxicities that would extend above Grade 2 after 6 months of follow-up. Early adverse events were also limited to Grade 2, including frequency and cystitis [10]. Similarly, Hass et al. reported only mild transient GU events and no significant GI toxicities [12]. The results of our study are consistent with previous studies, demonstrating an excellent safety profile for focal HDR BT, with only two cases of early Grade 2 GU toxicities (moderate dysuria and increased frequency). Additionally, no Grade 2 or higher GI toxicities were observed during the follow-up (Table 2). When our results are compared with focal LDR BT, it appears that focal HDR offers a similar toxicity profile. Langley et al. and Kunogi et al. demonstrated comparable rates of irritative GU symptoms and rectal discomfort, none exceeding grade 2 [14], 15]. However, LDR implants are associated with fixed–dose deposition and less flexible dosimetry, which may lead to increased GU and GI symptoms early on and later during follow-up [22], 23]. Moreover, in the whole-gland HDR BT series reported by Corkum et al. and Martinez et al., Grade 2 GU toxicities, such as urinary retention, dysuria, and frequency, were more common, ranging from 33.5 to 46.6 % [24], 25]. Although reported GI toxicities were less frequent, they were more prominent in papers reporting whole gland treatment results, reaching 8.6 %.

Results of PFS in our study suggest a potential benefit of focal HDR BT over AS for patients diagnosed with low- or favorable intermediate-risk PCa (mean PFS in months, respectively, 31.9 vs. 29.3, p=0.067), with 2-year PFS reaching 100 % in the focal HDR BT group. However, the mentioned trend toward improved PFS in the HDR group should be interpreted cautiously and considered exploratory, given the few progression events, the short median follow-up, and the borderline TNM imbalance. Nevertheless, the findings are comparable to those reported in other focal BT studies by Peters et al. (a 4-year BRFS of 70 %), Prada et al. (a 5-year BRFS of 62 %), and Hass et al. (a 2-year BRFS of 100 %). However, a study of whole gland BT published by Guinot et al. demonstrated better long-term disease control, with BRFS rates reaching 95.7 and 92.7 % at 5–10 years, respectively [26]. Similar results were published by Hudson et al., with an 8-year BDFS reaching 83.2 % [27]. However, as mentioned before, this benefit of better oncological control comes with the increased risk of toxicities related to the treatment, including urinary incontinence and rectal complications, which can persist for a long time and affect the QoL of patients. These improved oncological outcomes in whole-gland treatment compared to the focal treatment of PCa may be attributed to a few facts, such as PCa is multifocal [28], there is high heteroge-neity in foci of PCa [29], it is hard to detect low volume tumors within a standard 1.5 T MRI [30] and finally, in one out of eight patients, the satellite nodule will have a higher grade tumor than the index lesion [31].

Thus, focal HDR BT offers a compelling middle-ground strategy. While it may not achieve the same long-term oncologic control as whole-gland treatment, it can delay progression and extend the time without the morbidity associated with radical therapy. Compared with active surveillance, our results suggest that focal HDR may not only provide better PFS but also preserve quality of life, with urinary and functional outcomes remaining unaffected. This is particularly relevant for those patients who may experience anxiety over disease progression while being under AS. Moreover, focal therapy preserves radical treatment options such as prostatectomy or whole-gland BT for use at the time of progression, when disease burden justifies a more aggressive approach. This strategy supports the principle of risk-adapted, stepwise intervention in prostate cancer care, aiming to provide disease control with the least possible impact on quality of life while retaining the ability to escalate treatment when necessary.

Accordingly, the current findings of this study should be interpreted with caution and considered preliminary, as there are important limitations that must be acknowledged. Firstly, the sample size is relatively small (n=55), resulting in limited power. Furthermore, no formal adjustment for multiple comparisons was made. Therefore, findings should be interpreted cautiously due to the increased risk of Type I error and only after the long-term outcomes and recurrence data mature. Secondly, the limited follow-up duration of only 17 months may underestimate the incidence of late toxicities and recurrence patterns. Moreover, while the QoL outcomes were collected prospectively, not all patients reached the same follow-up time point. Thus, there are missing data at later time points, further reducing the statistical power and biasing the long-term assessment of the results. Additionally, oncological endpoints (biochemical/clinical progression) remain immature – adjusted time-to-event analyses are underpowered with only six recorded progression events, making current PFS results exploratory. Due to the poor accrual in the focal LDR group, this arm was not included in the interim analysis, thereby limiting comparisons across other focal therapy modalities. However, randomization remained the same 1:1:1 with concealed allocation. Inclusion of the LDR arm will allow a head-to-head comparison of patient-reported QoL and oncological control within the randomized setting. Lastly, as this is a single-center study, generalizability may be limited as it may introduce a potential center-specific practice effect. Although workflow was standardized and quality assurance protocols were used, confirmation in multicenter cohorts with longer follow-up is warranted.

However, there are several strengths, such as being one of the few prospective RCTs comparing focal HDR BT to AS in patients diagnosed with low- or favorable intermediate-risk PCa and evaluating the impact of focal therapy on QoL. Secondly, our RCT uses validated and disease-specific QoL instruments, such as the EORTC QLQ30, PR25, IPSS, and IIEF questionnaires, which were filled in consistently during the follow-up visits. Importantly, comparison with the AS group (control arm) provides an important benchmark for interpreting both QoL and oncological outcomes.

## Conclusions

A current study shows comparable QoL and early toxicity results between the AS and HDR groups. Any domain-level signals were below MCID and did not persist after baseline adjustment. Results of PFS are underpowered at this interim analysis due to short follow-up and high censoring, and should be considered hypothesis-generating only. However, as current results are only exploratory and insufficient to assess durable disease control or late toxicity, a longer follow-up, larger cohorts, and a multicenter approach are needed to provide definitive outcomes to demonstrate the benefit of focal brachytherapy.
